# The importance of the urea cycle and its relationships to polyamine metabolism during ammonium stress in *Medicago truncatula*

**DOI:** 10.1093/jxb/erac235

**Published:** 2022-05-24

**Authors:** Marina Urra, Javier Buezo, Beatriz Royo, Alfonso Cornejo, Pedro López-Gómez, Daniel Cerdán, Raquel Esteban, Víctor Martínez-Merino, Yolanda Gogorcena, Paraskevi Tavladoraki, Jose Fernando Moran

**Affiliations:** Institute for Multidisciplinary Research in Applied Biology (IMAB), Department of Sciences, Public University of Navarre (UPNA), Avda. de Pamplona 123, 31192 Mutilva, Spain; Institute for Multidisciplinary Research in Applied Biology (IMAB), Department of Sciences, Public University of Navarre (UPNA), Avda. de Pamplona 123, 31192 Mutilva, Spain; Institute for Multidisciplinary Research in Applied Biology (IMAB), Department of Sciences, Public University of Navarre (UPNA), Avda. de Pamplona 123, 31192 Mutilva, Spain; Institute for Advanced Materials and Mathematics (INAMAT2), Department of Sciences, Public University of Navarre (UPNA), Campus de Arrosadía, 31006 Pamplona, Spain; Institute for Multidisciplinary Research in Applied Biology (IMAB), Department of Sciences, Public University of Navarre (UPNA), Avda. de Pamplona 123, 31192 Mutilva, Spain; Institute for Multidisciplinary Research in Applied Biology (IMAB), Department of Sciences, Public University of Navarre (UPNA), Avda. de Pamplona 123, 31192 Mutilva, Spain; Department of Plant Biology and Ecology, University of the Basque Country (UPV/EHU), Sarriena s/n, Apdo. 644, 48080 Bilbao, Spain; Institute for Advanced Materials and Mathematics (INAMAT2), Department of Sciences, Public University of Navarre (UPNA), Campus de Arrosadía, 31006 Pamplona, Spain; Department of Pomology, Aula Dei Experimental Station, Consejo Superior de Investigaciones Científicas (CSIC), Avda. de Montañana 1005, 50059 Zaragoza, Spain; Department of Science, University Roma Tre, 00146 Rome, Italy; Institute for Multidisciplinary Research in Applied Biology (IMAB), Department of Sciences, Public University of Navarre (UPNA), Avda. de Pamplona 123, 31192 Mutilva, Spain; University of Birmingham, UK

**Keywords:** Amine oxidase, ammonium stress, nitrogen nutrition, polyamine, putrescine, urea cycle

## Abstract

The ornithine–urea cycle (urea cycle) makes a significant contribution to the metabolic responses of lower photosynthetic eukaryotes to episodes of high nitrogen availability. In this study, we compared the role of the plant urea cycle and its relationships to polyamine metabolism in ammonium-fed and nitrate-fed *Medicago truncatula* plants. High ammonium resulted in the accumulation of ammonium and pathway intermediates, particularly glutamine, arginine, ornithine, and putrescine. Arginine decarboxylase activity was decreased in roots, suggesting that the ornithine decarboxylase-dependent production of putrescine was important in situations of ammonium stress. The activity of copper amine oxidase, which releases ammonium from putrescine, was significantly decreased in both shoots and roots. In addition, physiological concentrations of ammonium inhibited copper amine oxidase activity in *in vitro* assays, supporting the conclusion that high ammonium accumulation favors putrescine synthesis. Moreover, early supplementation of plants with putrescine avoided ammonium toxicity. The levels of transcripts encoding urea-cycle-related proteins were increased and transcripts involved in polyamine catabolism were decreased under high ammonium concentrations. We conclude that the urea cycle and associated polyamine metabolism function as important protective mechanisms limiting ammonium toxicity in *M. truncatula*. These findings demonstrate the relevance of the urea cycle to polyamine metabolism in higher plants.

## Teaser

Metabolic changes of the urea cycle in connection to polyamine metabolism are an important protection mechanism during ammonium stress, revealing a role for the urea cycle in plants.

## Introduction

Ammonium (NH_4_^+^) as the sole source of nitrogen (N) is toxic for many plant species, leading to physiological and morphological disorders that affect plant growth and development (Britto and Kronzucker, 2002; Bittsánszky *et al.*, 2015; Esteban *et al.*, 2016a). Indeed, plants subjected to periods of high NH_4_^+^ availability develop imbalances of essential cations (Ariz *et al.*, 2011) and changes in the content of N-rich compounds, such as amino acids of low carbon/nitrogen (C/N) ratio (Ariz *et al.*, 2013; Vega-Mas *et al.*, 2019) and polyamines (PAs) (Houdusse *et al.*, 2005, 2008; Ariz *et al.*, 2013; Zhu *et al.*, 2018).

It has been proposed that the ornithine–urea cycle (urea cycle) might constitute an important control point of N metabolism in plants subjected to high NH_4_^+^ conditions (Moschou *et al.*, 2012; Esteban *et al.*, 2016a), since in lower photosynthetic eukaryotic phyla, the urea cycle significantly contributes to the metabolic responses under high N availability (Allen *et al.*, 2011). Nevertheless, the urea cycle is not considered complete in plants because the carbamoyl phosphate synthase-type I, the enzyme that synthesizes urea directly from NH_4_^+^, is absent. In contrast, plants possess a functional carbamoyl phosphate synthase-type II (CPSII, EC 6.3.5.5) that synthesizes urea from glutamine (Gln) instead of NH_4_^+^ (Zhou *et al.*, 2000; Brady *et al.*, 2010). In the urea cycle, the synthesis of arginine (Arg) from Gln is catalyzed by three sequential enzymes, ornithine transcarbamylase (OTC, EC 2.1.3.3), argininosuccinate synthase (AS, EC 6.3.4.5), and argininosuccinate lyase (AL, EC 4.3.2.1) (Micallef and Shelp, 1989; Slocum, 2005). Then, Arg is converted to ornithine (Orn) through the action of arginase (ARG, EC 3.5.3.1), with the concomitant production of urea (Kang and Cho, 1990; Winter *et al.*, 2015), and urea is further degraded by plant urease (URE, 3.5.1.5) to form NH_4_^+^ (Polacco and Winkler, 1984; Witte, 2011).

Furthermore, once Arg and Orn are produced in the urea cycle, the translocation of N towards the biosynthesis of the diamine putrescine (Put) occurs (see Esteban *et al.*, 2016a) through the consecutive action of the enzymes arginine decarboxylase (ADC, EC 4.1.1.19), agmatine iminohydrolase (AIH, EC 3.5.3.12), and *N*-carbamoyl-putrescine amidohydrolase (NCPAH, EC 3.5.1.53), and/or the enzyme ornithine decarboxylase (ODC, EC 4.1.1.17), respectively (Fuell *et al.*, 2010). The ADC pathway is present in plants but absent in metazoans (Slocum *et al.*, 1984), while the ODC pathway is considered to be universal (Michael *et al.*, 1996) except in some members of the Brassicaceae family, including *Arabidopsis thaliana* (Hanfrey *et al.*, 2001). Put is successively aminopropylated to form the triamine spermidine (Spd) in a reaction catalyzed by spermidine synthase (SPDS), and Spd is further converted to spermine (Spm) by the action of spermine synthase (SPMS) (Slocum *et al.*, 1984).

Plants finely regulate PA homeostasis at the level of synthesis, conjugation, turnover, transport, and catabolism (Moschou *et al.*, 2012; Tiburcio *et al.*, 2014). PA catabolism is mediated by numerous amine oxidases (AOs), including copper-containing amine oxidases (CuAOs, EC 1.4.3.6) and flavin-containing polyamine oxidases (PAOs, EC 1.5.3.3) (Cona *et al.*, 2006; Alcázar *et al.*, 2010; Wang *et al.*, 2019), with specific functions in plant tissue differentiation and development (Tavladoraki *et al.*, 2016). Additionally, PA catabolism contributes to several physiological processes as a source of stress-related molecules such as γ-aminobutyric acid (GABA) and hydrogen peroxide (H_2_O_2_). Particularly, PA-derived H_2_O_2_ contributes to generate oxidative stress within plant tissues or to activate antioxidative defense responses and cell-wall lignification (Su *et al.*, 2005; Angelini *et al.*, 2008; Gupta *et al.*, 2016).

Currently, there is little information on the overall effect of NH_4_^+^ on the urea cycle and PA metabolism, regardless of the remarkable accumulation of Put that NH_4_^+^ nutrition induces (Houdusse *et al.*, 2005, 2008; Belastegui-Macadam *et al.*, 2007; Ariz *et al.*, 2013; Zhu *et al.*, 2018). Indeed, there are four enzymatic steps releasing NH_4_^+^ and, therefore, NH_4_^+^ may act as a feedback regulator of these pathways. Filling in this knowledge gap could be essential to understand the onset of NH_4_^+^ stress, since the urea cycle–PA metabolism may represent a regulatory pathway in the management of N and, specifically, under NH_4_^+^ nutrition.

Here, we have analyzed the metabolite contents, transcript levels, and enzyme activities of the urea cycle and PA ­metabolism in *Medicago truncatula* plants growing in the presence of NH_4_^+^ as the only N source, in comparison to nitrate (NO_3_^–^)-fed plants. We found an accumulation of the intermediates Gln, Arg, Orn, and Put, an induction of the transcript levels of the urea-cycle-related genes and Put biosynthetic genes, and a decrease of CuAO activity during NH_4_^+^ stress, which highlighted the metabolic implications of these two interconnected routes. We have also addressed the question of whether Put led to the NH_4_^+^ stress or whether it is part of the mechanism of stress alleviation.

## Materials and methods

### Plant material and growth conditions

Seeds of *Medicago truncatula* Gaertn. ecotype Jemalong A17 were scarified with 95% sulfuric acid for 8 min, washed with sterile water, sterilized with 50% (v/v) sodium hypochlorite solution for 5 min, and consecutively washed with sterile water until the pH reached 7. After being kept overnight at 4 °C in darkness, seeds were germinated on Petri dishes containing 0.4% (w/v) plant agar for 72 h at 14 °C in darkness. Then, five sprouts were transferred to glass jars under axenic conditions, which contained 100 ml of modified Fahraeus media with 5 g l^–1^ of phytagel (Esteban *et al.*, 2016b; Buezo *et al.*, 2019). The growth medium contained 0.9 mM CaCl_2_, 0.5 mM MgSO_4_, 0.7 mM KH_2_PO_4_, 0.8 mM Na_2_HPO_4_, 20 μM ferric citrate, 0.8 μM MnCl_2_, 0.6 μM CuSO_4_, 0.7 μM ZnCl_2_, 1.6 μM H_3_BO_3_, 0.5 μM Na_2_MoO_4_, and 1 mM or 25 mM of N applied as either Ca(NO_3_)_2_ or (NH_4_)_2_SO_4_. Both 1 mM and 25 mM NH_4_^+^-fed plants were supplemented, respectively, with 0.5 mM and 12.5 mM CaSO_4_ to compensate for the Ca^2+^ supplied together with the NO_3_^–^ treatments.

To assess the effect of Put in the response to NH_4_^+^ nutrition, we supplemented plants with Put at 0.5 mM and we evaluated plant biomass as a marker of NH_4_^+^ stress. We used the following controls: a control with 2 mM NH_4_^+^ to discard the possibility that the Put effect relied partially on the concentration of N added to the culture medium, since adding Put implied the addition of an extra 1 mM of N; and a control with both 1 mM NH_4_^+^ and 1 mM NO_3_^–^ to assess whether the positive effect of exogenous Put was different from that of NO_3_^–^. No control was used at the high N concentration, as the difference between 25 mM and 26 mM of N was not considered relevant.

After a growth period of 14 d, shoots and roots were separately harvested, and both fresh weight and dry weight were measured. They were then frozen in liquid nitrogen, and stored at –80 °C for further analyses.

### Determination of inorganic soluble cation content

Ionic content was detected using a DIONEX-DX500 ion chromatograph equipped with an AS40 autosampler and ED40 electrochemical detector (Dionex Corporation, Sunnyvale, CA, USA) as described by Ariz *et al.* (2011). Frozen plant tissue (200 mg) was incubated in 1 ml of milli-Q water for 5 min at 80 °C in a water bath. The soluble ionic fraction was obtained by centrifugation at 16 000 *g* for 30 min. The supernatants, stored at –20 °C, were diluted 1:10 for injection. Ion Pac CG12A and Ion Pac CG12A were used as the stationary phase and 30% 100 mM NaOH and 70% milli-Q water were used as the mobile phase at 1.5 ml min^–1^ flow rate for 15 min. Soluble cations (Na^+^, K^+^, Mg^2+^, Ca^2+^, and NH_4_^+^) were determined using 20 mM methanosulfonic acid as the mobile phase for 13 min.

### Determination of amino acid and protein content

Determination of amino acids was performed using high-performance capillary electrophoresis in a Beckman Coulter PA-800 (Coulter Inc., Brea, CA, USA) equipped with a laser-induced fluorescence detector (argon ion: 488 nm) as described in Ariz *et al.* (2013). Frozen plant tissue (100 mg) was ground with a mortar and pestle using liquid nitrogen, homogenized with 1.5 ml of 1 M HCl, incubated in ice for 10 min, and centrifuged at 13 000 *g* for 10 min at 4 °C. The recovered supernatants were neutralized with NaOH and stored at –80 °C. Amino acids were derivatized with fluorescein isothiocyanate dissolved in 20 mM ­acetone/borate, pH 10, at room temperature for 12–16 h. Samples were injected in a migration buffer composed of 80 mM borax and 45 mM α-cyclodextrin, pH 9.2, using a pressurized method (5 s, 3.45 kPa). Single amino acids were eluted in a 50 µm internal diameter × 43/53.2 cm fused silica capillary at a voltage of 30 kV and 20 °C. Norvaline and homoglutamic acid were used as internal standards. Protein content was measured using a dye-binding Bradford microassay (Bio-Rad, Watford, UK) with bovine serum albumin as a standard.

### Determination of polyamine content

The content of free PAs was measured using a Waters 575 High-Performance Liquid Chromatography Pump controlled by a Waters Pump Control Module and equipped with a Waters 474 fluorescence detector (Waters, Milford, MA, USA) as described in Ariz *et al.* (2013) with some modifications. Frozen plant tissue (200 mg) was ground with liquid nitrogen and homogenized with 10:1 (v/w) of extraction buffer (5% aqueous HClO_4_, w/w):plant tissue, containing 0.1:1 (v/w) of 2 mM 1,6-hexanediamine:plant tissue as an internal standard solution. Samples were shaken at 24 000 rpm for 15 s, incubated for 1 h at 4 °C, and centrifuged at 15 000 *g* for 15 min at 4 °C. Volumes of 200 µl of the recovered supernatant were mixed with 400 µl of 3 M aqueous Na_2_CO_3_ and 400 µl of 0.12 M dansyl chloride in acetone, and incubated in darkness for 1 h at 60 °C. The reaction was quenched by the addition of 100 µl of 0.87 M proline and further incubation for 30 min. Then, PAs were extracted using 5 ml of ethyl acetate. Samples were centrifuged at 3000 *g* for 5 min at room temperature, organic layers were recovered, and the solvent was removed under reduced pressure at 40 °C. PAs were dissolved in 0.5 ml of methanol and filtered with a 0.45 µm pore nylon filter. The content of PAs was determined from 25 µl of extract. A Tracer Excel 120-ODSA column (3 µm 4.6 × 150 mm, Teknokroma, Barcelona, Spain) at 30 °C was used as the stationary phase. The mobile phase consisted of solvent A (water) and B (methanol), with a constant flux of 0.5 ml min^–1^. An increasing concentration gradient was used for solvent B, from 58% to 100% over 44 min, and then remained constant for 4 min. The concentration of solvent B was then gradually decreased to 58% for 3 min and allowed to rest for an additional 3 min. The fluorescence detector was set at λ_ex_=350 nm, λ_em_=515 nm. Retention times were 29.88 min for Put, 45.06 min for Spd, and 48.85 min for Spm.

### Determination of ADC and OCD enzymatic activities

The activities of ADC and ODC enzymes were determined according to Wu *et al.* (2012). Frozen plant tissue (200 mg) was ground with liquid nitrogen and homogenized with 1.5 ml of 0.1 M cold phosphate buffer, pH 6.3, which contained 5 mM EDTA, 1 mM pyridoxal phosphate, 0.01 mM polyvinyl pyrrolidone, 10 mM dithiothreitol, and 0.43 mM sodium thiosulfate. The homogenates were centrifuged at 12 000 *g* for 40 min at 4 °C. A volume of 0.8 ml of the recovered supernatant was mixed with 1 ml of reaction mix, composed of 0.1 M Tris–HCl buffer, pH 7.5, 5 mM EDTA, 40 μM pyridoxal phosphate, and 5 mM dithiothreitol, and 0.2 ml of either 25 mM l-Arg (for ADC activity) or 25 mM l-Orn (for ODC activity). To calculate the specific activity, control samples in which l-Arg or l-Orn were replaced by perchloric acid were used. A volume of 0.5 ml of the sample was mixed with 1 ml of 2 M NaOH and 10 μl benzoyl chloride, stirred for 20 s, and incubated for 30 min at 37 °C. A volume of 2 ml of saturated NaCl and 2 ml of 100% ether was added and the mixture was centrifuged at 1500 *g* for 5 min at 4 °C. Subsequently, 1 ml of the ether phase extraction was evaporated at 50 °C in a water bath, and the remainder was dissolved in 3 ml of 100% methanol. The reaction was measured at 254 nm in a GeneQuant 1300 spectrophotometer (Harvard Bioscience Inc., Holliston, MA, USA). An increase in absorbance of 1.0 at 254 nm for 1 min was considered one activity unit of enzyme.

### Determination of CuAO and PAO enzymatic activities

Amine oxidase activity was determined as described by Su *et al.* (2005) with some modifications. Plant frozen tissue (200 mg) was ground with liquid nitrogen and homogenized with 2:1 (v/w) 100 mM sodium phosphate buffer, pH 6.5. The homogenates were centrifuged at 12 000 *g* for 20 min at 4 °C. A volume of 10 µl of the recovered supernatant was mixed with the reaction mix, composed of 10 µl of 15 mM 4-aminoantipyrine/0.2% (v/v) *N*,*N*-dimethylaniline, 250 U ml^–1^ horseradish peroxidase, and 20 mM PAs. The AO activity was assayed using Put, Spd, or Spm as substrate. To calculate the specific activity, control samples without PAs were used. The reaction was measured at 555 nm in a SpectraMax 340pc microplate reader (Molecular Devices, San Jose, CA, USA), after being incubated at room temperature for 30 min. A change of 0.01 in absorbance was considered one activity unit of AO enzyme (Su *et al.*, 2005).

To calculate the inhibition parameters of CuAO activity by NH_4_^+^, increasing concentrations of (NH_4_)_2_SO_4_ from 1 mM to 100 mM were added to the reaction mixture. The maximum rate (*V*_max_) and Michaelis constant (*K*_m_) values were determined from Michaelis–Menten plots, and the inhibitor constant (*K*_i_) values were obtained from a non-linear regression fitting of data for a mixed inhibition model performed with GraphPad Prism (version 8.4.0).

### Determination of H_2_O_2_ content

The content of H_2_O_2_ was quantified using an Amplex Red H_2_O_2_ assay according to the manufacturer’s instructions (Invitrogen, Carlsbad, CA, USA). Frozen plant tissue (70 mg) was ground with liquid nitrogen and homogenized with 4:1 (v/w) 50 mM sodium phosphate buffer, pH 7.4. Samples were centrifuged at 12 000 *g* for 20 min at 4 °C. Volumes of 15 µl of plant extract were mixed with 85 µl of reaction mix, composed of 25 µM Amplex Red reagent and 0.2 U ml^–1^ horseradish peroxidase. The mixture was shaken at 450 rpm for 1 min and then incubated for 50 min at 30 °C. Samples were read every 10 min to follow the reaction kinetics at 570 nm in a SpectraMax 340pc microplate reader (Molecular Devices, San Jose, CA, USA). An H_2_O_2_ standard curve was prepared to quantify the H_2_O_2_ content of every sample.

### Gene identification, RNA isolation, and gene expression analyses

The selection of urea cycle- and PA-related genes was done in the following databases linked to the *M. truncatula* genome (Tang *et al.*, 2014): GenBank, Phytozome, Uniprot, and QuickGO. In total, 25 genes encoding for CPSII (*Medtr4g103830* and *Medtr2g093280*), OTC (*Medtr1g022420* and *Medtr3g112050*), AS (*Medtr3g088970* and *Medtr5g042880*), AL (*Medtr3g100220*), ARG (*Medtr4g024960*), URE (*Medtr3g085640*), ODC (*Medtr3g114870*), ADC (*Medtr3g113910* and *Medtr4g072020*), AIH (*Medtr4g112810*), NCPAH (*Medtr2g086600*), CuAO (*Medtr1g104590*, *Medtr4g117610*, *Medtr3g080500*, *Medtr3g077080*, *Medtr5g033170*, *Medtr8g069505*, and *Medtr1g104550*), and PAO (*Medtr3g033000*, *Medtr5g090300*, *Medtr3g064370*, and *Medtr2g039160*) were studied.

RNA was extracted from 100 mg of frozen plant tissue powder using TRIzol^®^ reagent (Invitrogen, Carlsbad, CA, USA) and RQ1 RNase-free DNase (Promega, Madison, WI, USA) according to the manufacturer’s instructions. The concentration and integrity of RNA were verified by the 260 nm/280 nm absorption ratio in an ND-1000 spectrophotometer (Thermo Scientific, Waltham, MA, USA). RNA (1 µg) was retrotranscribed into cDNA with the PrimeScriptTM RT kit (Takara Bio Inc., Kusatsu, Shiga, Japan).

The specific primers (Supplementary Table S1) were designed using the Primer-BLAST bioinformatics tool. Gene expression was determined from 2 µl of cDNA diluted 1:5 in a 20 µl reaction volume using SYBR^®^ Premix Ex Taq^TM^ (Takara Bio Inc., Kusatsu, Shiga, Japan) in a Step One Plus Real Time PCR System (Applied Biosystems, Foster City, CA, USA) as described in Buezo *et al.* (2019). The PCR program was 95 °C for 5 min, 40 cycles of 15 s at 94 °C followed by 1 min at 60 °C, and a final melting curve was programmed to confirm the absence of contamination with genomic DNA. Ubiquitin carrier protein 4 (*Medtr3g062450*) and 26S proteasome regulatory subunit S5A_2 (*Medtr5g022440*) were used as *M. truncatula* reference genes (Larrainzar *et al.*, 2015).

### Phylogenetic analysis

The amino acid sequences of CuAOs and PAOs were retrieved by a BLASTp analysis using *A. thaliana* orthologous proteins as query sequences to retrieve those of *M. truncatula* by sequence similarity. Multiple amino acid sequence alignments were performed using the MAFFT 7 E-INS-I algorithm, which included the characterized orthologous proteins from *A. thaliana* (Møller and McPherson, 1998; Tavladoraki *et al.*, 2006; Takahashi *et al.*, 2010; Fincato *et al.*, 2011; Wimalasekera *et al.*, 2015, 2011; Planas-Portell *et al.*, 2013; Ghuge *et al.*, 2015; Qu *et al.*, 2017; Fraudentali *et al.*, 2020; Liu *et al.*, 2020), *Lens culinaris* (Rossi *et al.*, 1992), *Malus domestica* (Zarei *et al.*, 2015), *Oryza sativa* (Ono *et al.*, 2012; Liu *et al.*, 2014), and *Pisum sativum* (Tipping and McPherson, 1995; Laurenzi *et al.*, 2001; Petřivalský *et al.*, 2007; Sagor *et al.*, 2021) (Supplementary Table S2). The resulting phylogenetic tree was obtained through the neighbor-joining method and the statistical bootstrap test with 1000 replications (Katoh *et al.*, 2019) and plotted by using iTOL (Letunic and Bork, 2021).

### Statistical analysis

All statistical analyses were conducted using R studio (version 3.6.2) (R Core Team, 2020). Independent replicates (*n*=3–8) of each treatment were analyzed in the experiments. All data were tested for normality with the Shapiro–Wilk test and for homogeneity of variances with the Bartlett test. Differences among treatments were evaluated with one-way ANOVA and post hoc Student–Newman–Keuls test. For non-parametric data, Welch’s ANOVA and Games–Howell tests were performed. Differences were considered statistically significant at *P*<0.05.

## Results

### High NH_4_^+^ nutrition led to NH_4_^+^ over-accumulation but did not affect K^+^ concentration in *M. truncatula* plants

**Fig. 1.**
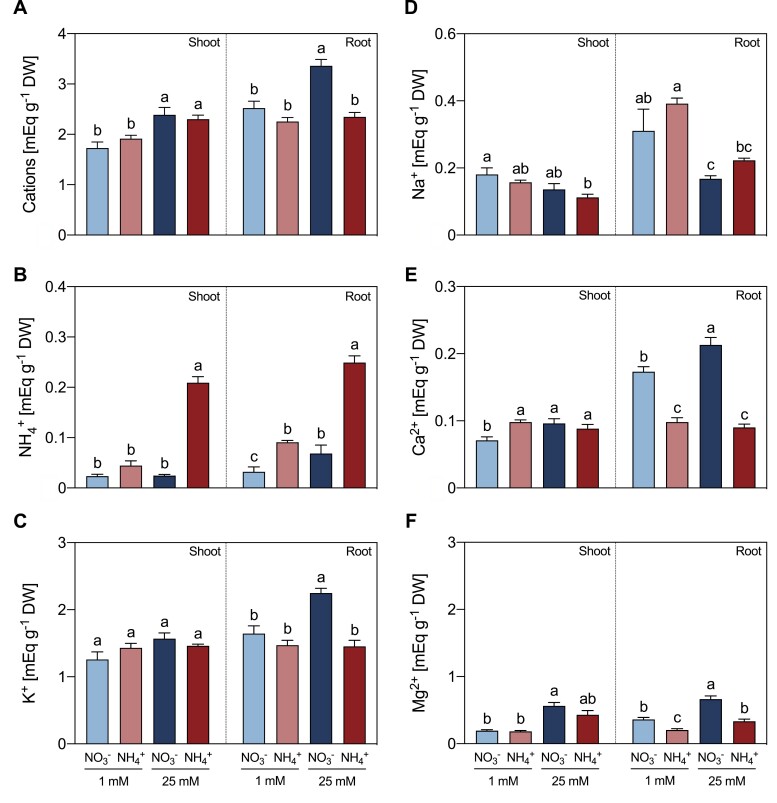
Effect of different sources and concentrations of N nutrition on the content of total soluble cations (A), NH_4_^+^ (B), K^+^ (C), Na^+^ (D), Ca^2+^ (E), and Mg^2+^ (F) in shoots and roots of 14-day-old *M. truncatula* seedlings. Data represent means ±SE values (*n*=4). Different letters denote statistically significant differences at *P*<0.05.

Plants supplied with high doses of either NO_3_^–^ or NH_4_^+^ exhibited the highest content of soluble cations in shoots, whereas solely 25 mM NO_3_^–^ nutrition increased the total soluble cation content in roots (Fig. 1A). At the shoot level, 25 mM NH_4_^+^ nutrition led to a large increase in NH_4_^+^ internal content in comparison with the 1 mM and 25 mM NO_3_^–^ treatments (8.9- and 8.5-fold, respectively). A similar increase in NH_4_^+^ internal content was found in roots (7.7- and 3.6-fold, respectively). Besides, the NH_4_^+^ content in roots was greater in plants fed 1 mM NH_4_^+^ than 1 mM NO_3_^–^ plants (2.8-fold) (Fig. 1B).

Regarding essential cations, the K^+^ content was higher in roots of 25 mM NO_3_^–^-fed plants than in roots of 1 mM NO_3_^–^-fed plants, while it remained unchanged in 1 mM or 25 mM NH_4_^+^-fed plants relative to 1 mM NO_3_^–^-fed plants (Fig. 1C). The Na^+^ content decreased in both shoots and roots as the N dose increased, but no significant differences in Na^+^ content were detected among plants grown with different N sources (Fig. 1D). Moreover, Ca^2+^ and Mg^2+^ contents were lower in roots of NH_4_^+^-fed plants than in roots of NO_3_^–^-fed plants (Fig. 1E, F).

### NH_4_^+^ nutrition highly induced the accumulation of the urea cycle metabolites Gln, Arg, and Orn and the diamine Put

The highest content of total amino acids was found in both shoots and roots of 25 mM NH_4_^+^-fed plants. By contrast, the soluble protein content increased only in shoots of 25 mM NH_4_^+^-fed plants in comparison with NO_3_^–^-fed plants (Supplementary Fig. S1).

**Fig. 2.**
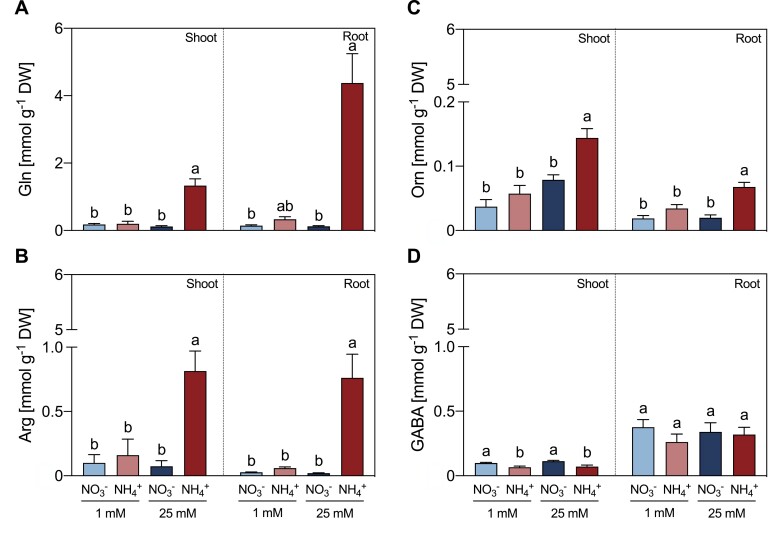
Effect of different sources and concentrations of N nutrition on the urea-cycle-related amino acids Gln (A), Arg (B), Orn (C), and GABA (D) in shoots and roots of 14-day-old *M. truncatula* seedlings. Data represent means ±SE values (*n*=4). Different letters denote statistically significant differences at *P*<0.05.

Gln content was highly increased in shoots and roots of 25 mM NH_4_^+^-fed plants (7.6- and 30.6-fold, respectively) compared with NO_3_^–^-fed plants (Fig. 2A; Supplementary Fig. S2). Furthermore, the contents of Arg and Orn, pivotal N compounds within the urea cycle and precursors of PAs, increased remarkably in shoots (8.2- and 3.9-fold, respectively) and roots (28.4- and 3.6-fold, respectively) of plants subjected to high NH_4_^+^ nutrition relative to plants grown in the presence of 1 mM NO_3_^–^ (Fig. 2B, C). By contrast, NH_4_^+^ treatment at both concentrations caused a 30% decrease in the content of the non-protein amino acid GABA in shoots, but not in roots (Fig. 2D).

**Fig. 3.**
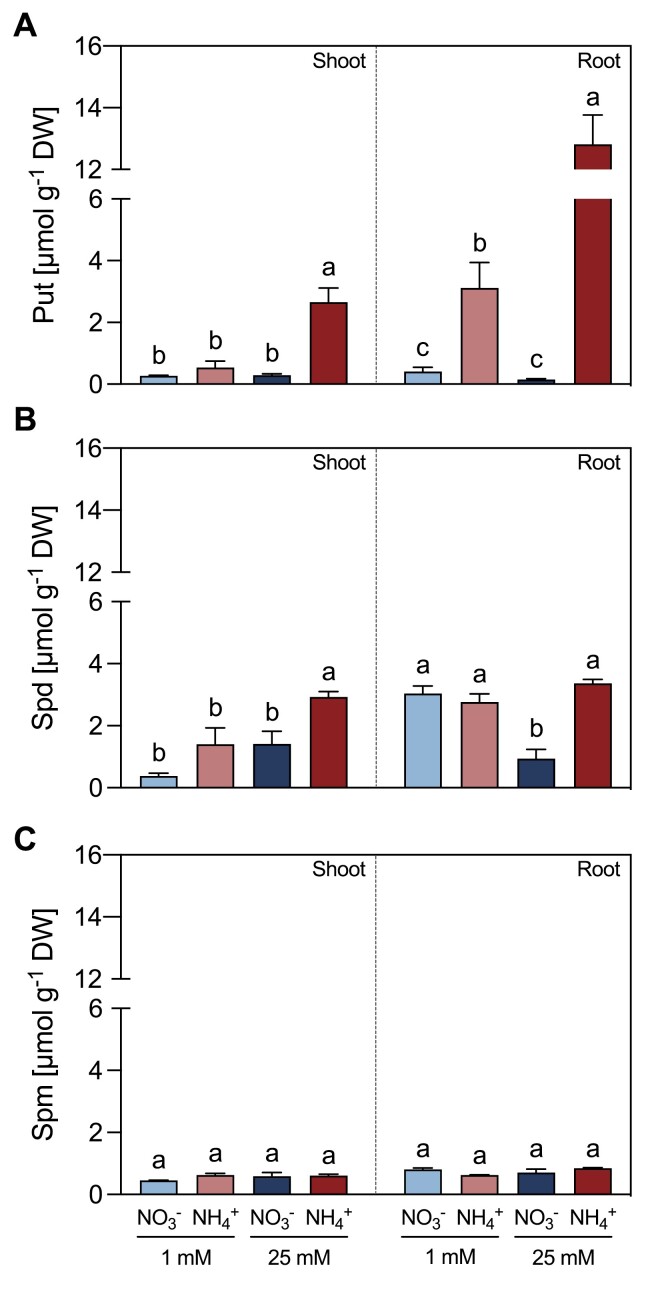
Effect of different sources and concentrations of N nutrition on the content of the polyamines Put (A), Spd (B), and Spm (C) in shoots and roots of 14-day-old *M. truncatula* seedlings. Data represent means ±SE values (*n*=3). Different letters denote statistically significant differences at *P*<0.05.

Plants grown under 25 mM NH_4_^+^ showed 9.2- and 2.1-fold larger contents of Put (Fig. 3A) and Spd (Fig. 3B) in shoots, respectively, compared with 25 mM NO_3_-fed plants. Similarly, roots of 1 mM and 25 mM NH_4_^+^-fed plants showed significantly higher Put content than NO_3_^–^-fed plants (7.6- and 87.2-fold, respectively) (Fig. 3A). No significant differences in Spm content were observed between NO_3_^–^- and NH_4_^+^-fed plants (Fig. 3C).

### NH_4_^+^ nutrition decreased ADC and CuAO activities, but induced ODC activity

**Fig. 4.**
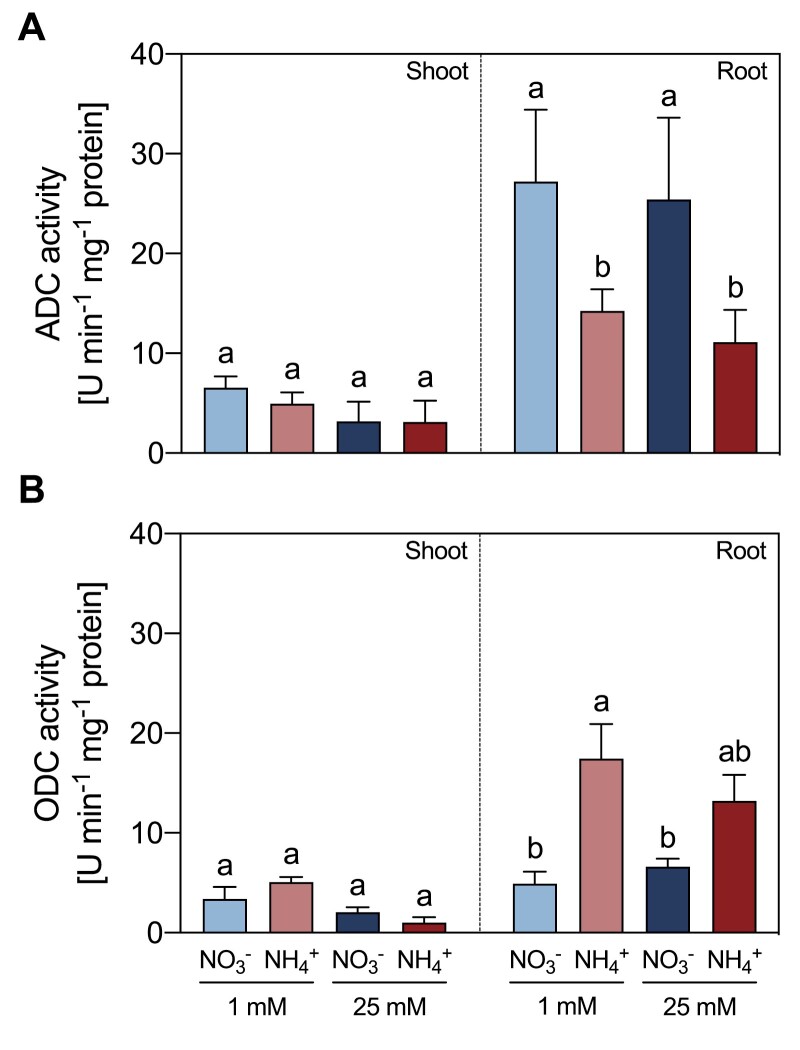
Effect of different sources and concentrations of N nutrition on ADC (A) and ODC (B) enzymatic activity in shoots and roots of 14-day-old *M. truncatula* seedlings. The enzymatic activity is expressed in enzyme units min^–1^ mg^–1^ protein. Arg was used as substrate in the ADC assay, while Orn was used as the substrate for ODC activity. Data represent means ±SE values (*n*=3–4). Different letters denote statistically significant differences at *P*<0.05.

**Fig. 5.**
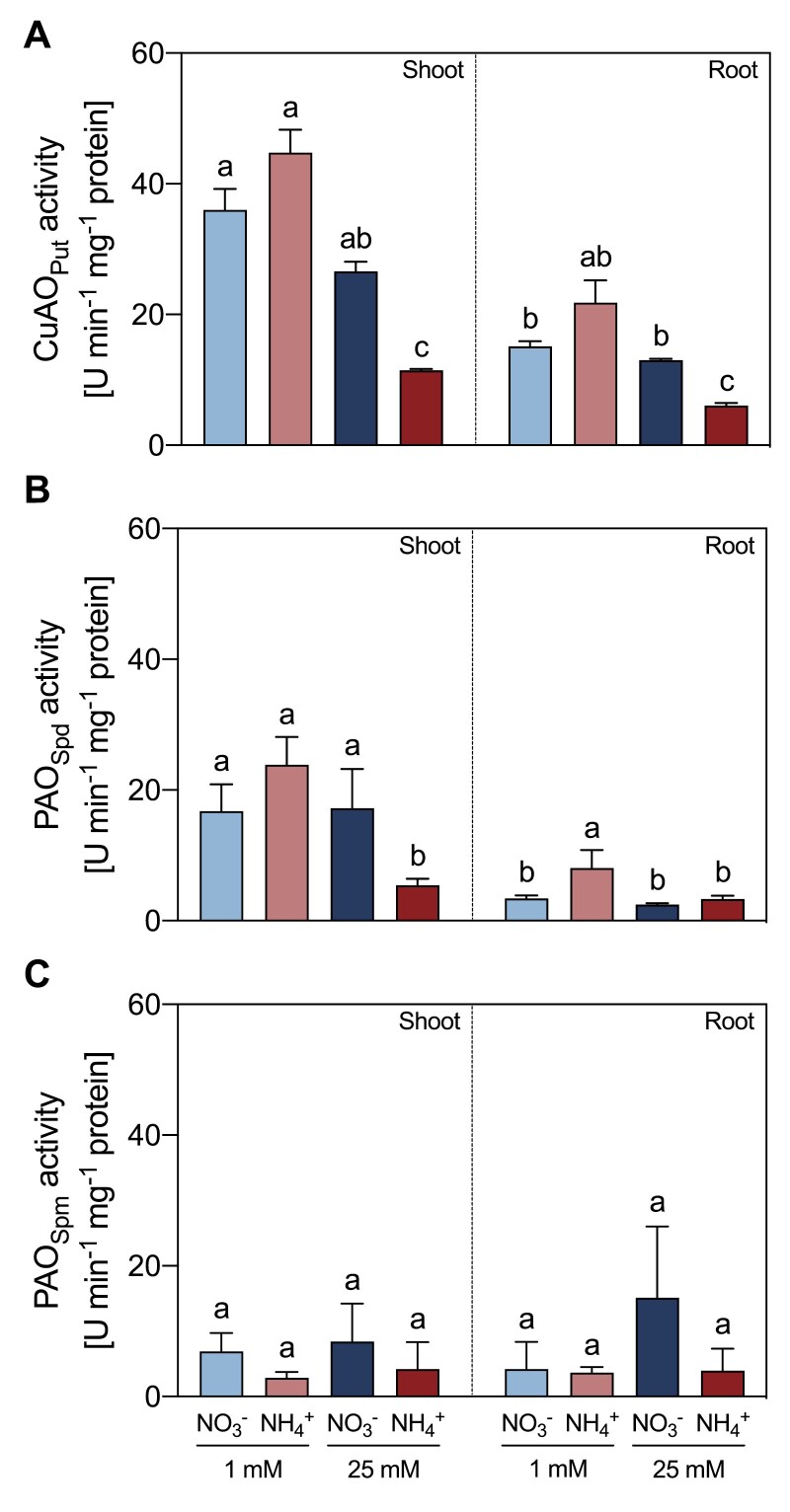
Effect of different sources and concentrations of N nutrition on Put-dependent CuAO (A), Spd-dependent PAO (B), and Spm-dependent PAO (C) enzymatic activity in shoots and roots of 14-day-old *M. truncatula* seedlings. The enzymatic activity is expressed in enzyme units min^–1^ mg^–1^ protein. Put was used as the substrate in the CuAO assay (A), while Spd (B) or Spm (C) were used as substrates for PAO activity. Data represent means ±SE values (*n*=3). Different letters denote statistically significant differences at *P*<0.05.

In roots, NH_4_^+^-fed plants showed a decrease in ADC activity at both NH_4_^+^ concentrations, relative to NO_3_^–^-fed plants (Fig. 4A). By contrast, ODC activity increased in both 1 mM and 25 mM NH_4_^+^-fed plants, respectively (Fig. 4B). No significant differences in the activity of either enzyme were observed in shoots (Fig. 4A, B). The Put-dependent AO activity was significantly lower by 57% in shoots and 53% in roots of 25 mM NH_4_^+^-fed plants compared with 25 mM NO_3_^–^-fed plants (Fig. 5A). Spd-dependent PAO activity was also lower in shoots of 25 mM NH_4_^+^-fed plants in comparison to 25 mM NO_3_^–^-fed plants, whereas no significant difference between the two treatments was observed in roots (Fig. 5B). Spm-dependent PAO activity did not vary significantly between tissues or treatments (Fig. 5C).

**Fig. 6.**
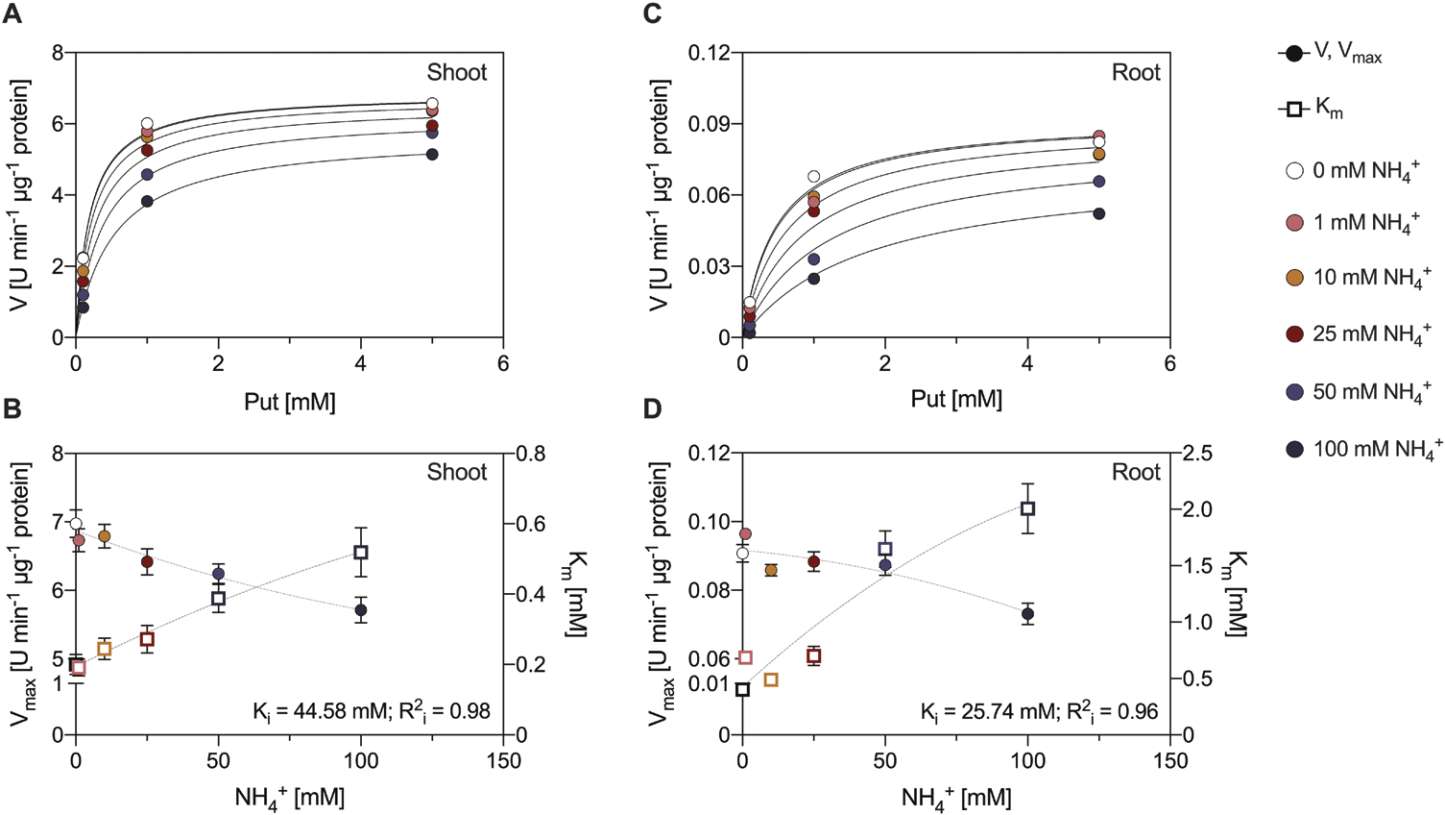
Inhibitor constant (*K*_i_) of NH_4_^+^ on CuAO activity in extracts of *M. truncatula* shoots (A, B) and roots (C, D) grown under 1 mM NO_3_^–^ for 14 days. Michaelis–Menten plots were obtained using Put as the substrate and NH_4_^+^ as an inhibitor (A, C). Maximum rate (*V*_max_) and Michaelis constant (*K*_m_) are plotted against the concentration of inhibitor from 0 to 100 mM NH_4_^+^ (B, D). Data represent means ±SE values (*n*=3).

To test whether NH_4_^+^ may act as a feedback regulator of Put oxidation, we analyzed *in vitro* the AO activity in the presence of NH_4_^+^. The inhibition studies with NH_4_^+^ evidenced that CuAO activity was competitively affected when the NH_4_^+^ concentration was <50 mM, since *V*_max_ remained unchanged while the *K*_m_ increased. The measured *K*_i_ for NH_4_^+^ was 44.6 mM in shoots (Fig. 6A, B) and 25.7 mM in roots (Fig. 6C, D). From the fresh and dry weight data we could estimate a water content of 90% for shoots and 94% for roots. The internal concentration of NH_4_^+^ in the root ranged around 25 mM (Fig. 1), which was also in the range of the *K*_i_ measured for NH_4_^+^ in roots. These results evidenced that CuAO ­activities were inhibited by the increase of internal NH_4_^+^ content, especially in 25 mM NH_4_^+^-fed plants.

**Fig. 7.**
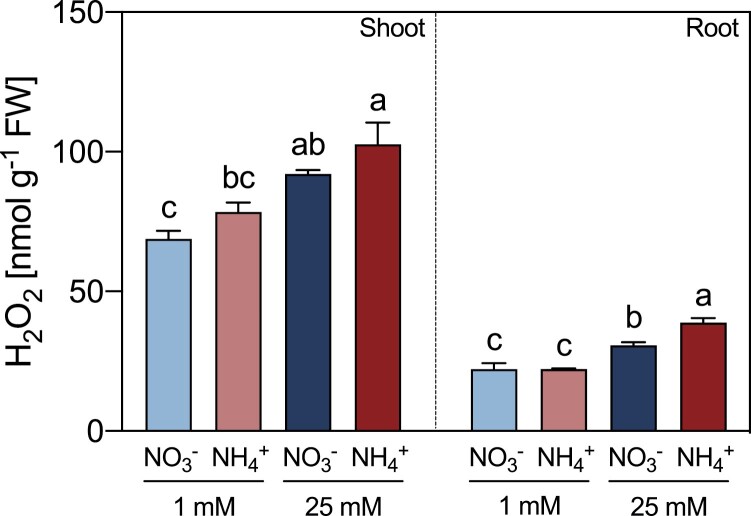
Effect of different sources and concentrations of N nutrition on H_2_O_2_ content in shoots and roots of 14-day-old *M. truncatula* seedlings. Data represent means ±SE values (*n*=3). Different letters denote statistically significant differences at *P*<0.05.

The H_2_O_2_ content was measured since H_2_O_2_ is one of the AO reaction products. Both shoots and roots of *M. truncatula* plants exhibited a significant increase in H_2_O_2_ content under conditions of high N, with the greatest increase being observed in plants grown in the presence of 25 mM NH_4_^+^ (Fig. 7). These results contrasted the reduction of AO activity under conditions of high NH_4_^+^ (Supplementary Fig. S3). Thus, it is possible that other H_2_O_2_-producing enzymes, different from CuAOs and PAOs, contribute to the increase of H_2_O_2_ content under high NH_4_^+^ conditions.

**Fig. 8.**
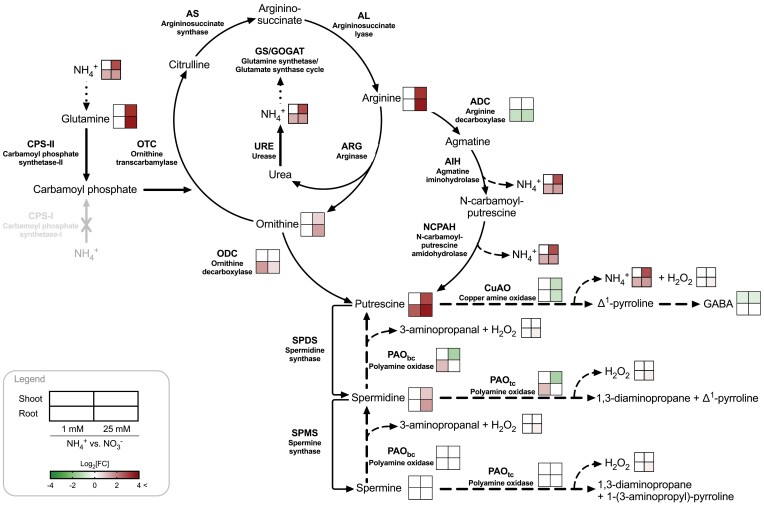
The urea cycle and PA metabolism in plants. Enzymes are represented in bold text, and metabolites in regular text (after Esteban *et al.*, 2016a). Changes, when comparing NH_4_^+^ versus NO_3_^–^ nutrition, are depicted in green when either metabolite content or enzymatic activity decreased, and in red when they increased. Shoot and root tissues are represented by the upper and lower squares, respectively. Illustration created with BioRender 2022.

Taken together, these results demonstrated that NH_4_^+^ nutrition at a high dose had important effects on the content of many of the urea cycle–PA metabolism intermediates (i.e. Gln, Arg, Orn, and Put) as well as the activities of the enzymes (i.e. ADC, ODC, and CuAO), in comparison with plants grown under a high dose of NO_3_^–^ (Fig. 8).

### NH_4_^+^ nutrition modulated the transcript levels of genes involved in the urea cycle and polyamine metabolism in *M. truncatula* plants

**Fig. 9.**
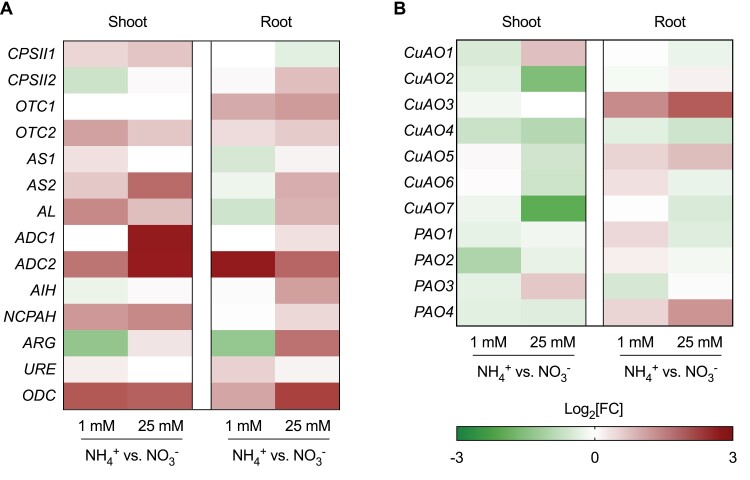
Effect of different concentrations of NH_4_^+^ nutrition on the transcript levels of the genes involved in the urea cycle and Put biosynthesis (A) and PA catabolism (B) assayed from RNA samples of 14-day-old *M. truncatula* seedlings (*n*=3–4). Data were calculated relative to transcript levels in seedlings exposed to 1 mM and 25 mM NO_3_^–^ treatments, respectively.

In both shoots and roots, 1 mM and 25 mM NH_4_^+^ nutrition increased the transcript levels of the urea cycle genes *MtOTC2*, *MtAS2*, and *MtAL*, in comparison to NO_3_^–^ nutrition at the respective doses. Moreover, transcript levels of *MtARG* were diminished by NH_4_^+^ treatment at a low dose but increased by 25 mM NH_4_^+^ nutrition compared with NO_3_^–^. No changes were observed in the expression level of *MtURE*. Transcript levels of the genes *MtADC1*, *MtADC2*, and *MtODC*, involved in Put biosynthesis, were strongly increased in plants grown under NH_4_^+^ conditions relative to NO_3_^–^-fed plants in both shoot and root tissues (Fig. 9A).

**Fig. 10.**
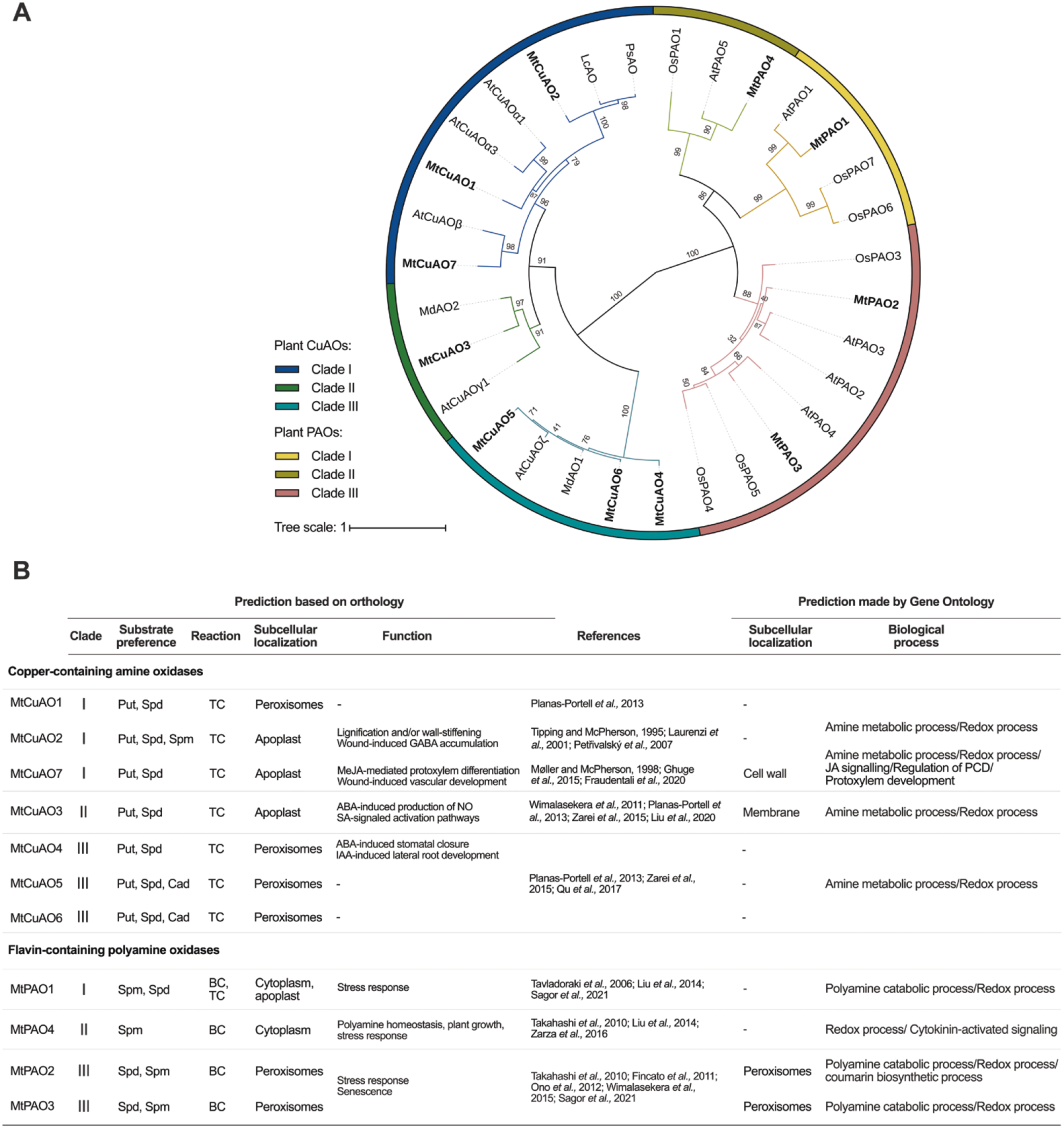
Interspecific phylogenetic tree of *M. truncatula* copper amine oxidases and polyamine oxidases. Amino acid sequences were aligned with the MAFFT 7 E-INS-I algorithm. The phylogenetic tree was performed by using the neighbor-joining method and the statistical bootstrap test with 1000 replications. The obtained bootstrap values are indicated at the nodes (A). The predicted substrate preference, involvement in back-conversion (BC) or terminal catabolism (TC), subcellular localization, and function of each enzyme based on the already characterized orthologous genes, and the prediction made by the Gene Ontology Annotation database (B).

Regarding genes involved in PA catabolism, in the *M. truncatula* genome seven *CuAO* genes have been identified in *in silico* analyses. An interspecific phylogenetic tree generated from predicted amino acid sequences indicated that MtCuAO1, MtCuAO2, and MtCuAO7 belonged to Clade I (Tavladoraki *et al.*, 2016) together with AtCuAOα1, AtCuAOα3, AtCuAOβ, LcAO, and PsAO. MtCuAO3 was grouped in Clade II together with AtCuAOγ1 and MdAO2. MtCuAO4, MtCuAO5, and MtCuAO6 belonged to Clade III together with AtCuAOζ and MdAO1 (Fig. 10A, B).

Four *PAO* genes were present. In particular, MtPAO1 belonged to Clade I of the PAO phylogenetic tree (Salvi and Tavladoraki, 2020) along with AtPAO1, OsPAO6, and OsPAO7. MtPAO4 was grouped in Clade II together with AtPAO5 and OsPAO1. MtPAO2 and MtPAO3 belonged to Clade III along with AtPAO2, AtPAO3, AtPAO4, OsPAO3, OsPAO4 and OsPAO5 (Fig. 10A, 10B).

Transcript levels of *MtCuAO2* and *MtCuAO7* were lower in shoots of 25 mM NH_4_^+^-fed plants compared with 25 mM NO_3_^–^-fed plants. In contrast, *MtCuAO1* and *MtPAO3* transcript levels were higher in these plants. In roots, transcript ­levels of CuAOs were slightly lower in 25 mM NH_4_^+^-fed plants, except for *MtCuAO3* and *MtCuAO5*, which were higher in comparison to plants grown under 25 mM NO_3_^–^; the same pattern was also observed for *MtPAO4* (Fig. 9B).

### Exogenous supplementation with Put improved biomass accumulation under NH_4_^+^ nutrition

**Fig. 11.**
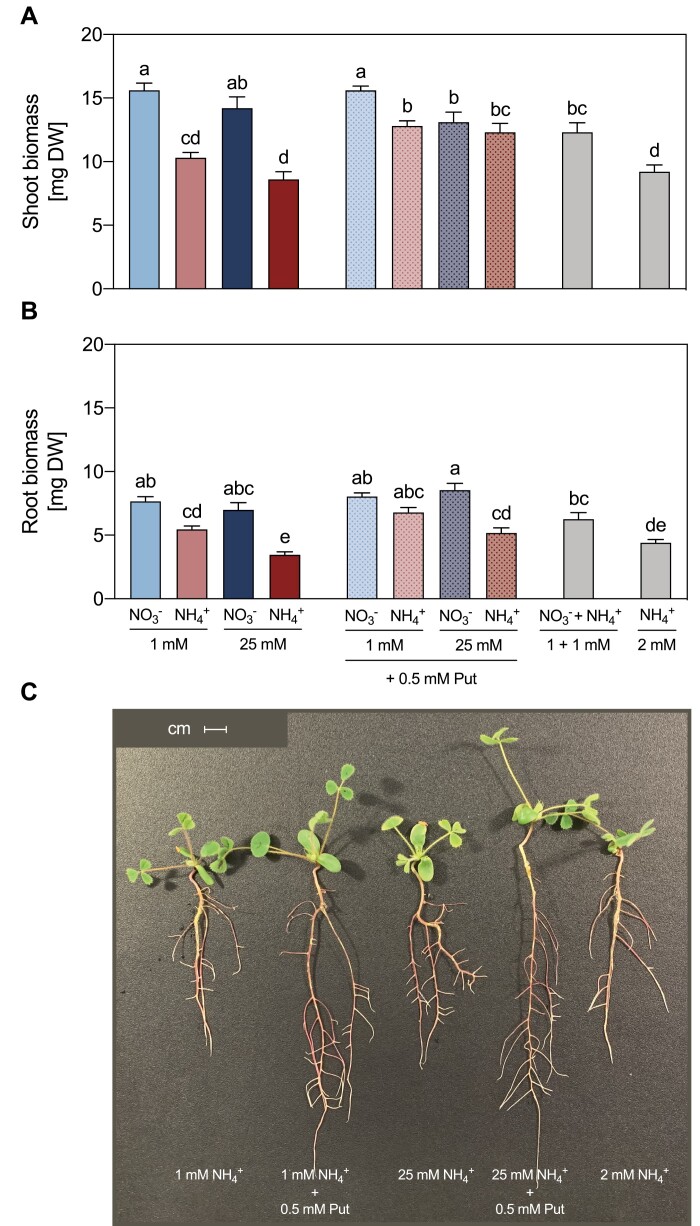
Effect of different sources and concentrations of N nutrition on the dry biomass of 14-day-old *M. truncatula* seedlings. (A, B) Distribution of plant biomass of shoots (A) and roots (B) expressed as dry weight (DW) per plant subjected to different N treatments and supplemented with Put. Data represent means ±SE values (*n*=8). Different letters denote statistically significant differences at *P*<0.05. Representative image of NH_4_^+^-fed plants (C). Scale bar=1 cm.

The growth of *M. truncatula* plants in the presence of NH_4_^+^ appeared strongly reduced compared with the growth in the presence of NO_3_^–^. Indeed, biomass production in NH_4_^+^-fed plants was lower than that in NO_3_^–^-fed plants. At low dose, NH_4_^+^-fed plants had 34% and 29% lower shoot (Fig. 11A) and root (Fig. 11B) dry biomass, respectively, than NO_3_^–^-fed plants. At high dose, the decrease in the dry biomass of NH_4_^+^-fed plants relative to NO_3_^–^-fed plants was 40% in shoots and 51% in roots. The addition of Put to the NH_4_^+^-containing medium alleviated the reduction in plant growth (Fig. 11C), whereas it did not affect plant growth when added to the NO_3_^–^-containing nutrient solution. The effect of Put in plants grown in NH_4_^+^-containing medium was observed at both shoot and root levels, as shown by measuring shoot and root dry weight (Fig. 11). Adding Put to the growth medium induced a similar effect on plant biomass than that of 1 mM NO_3_^–^ supplementation in the nutrient solution. As Put at 0.5 mM represented an additional 1 mM of N supplemented to the plant, we included a 2 mM NH_4_^+^ control. The Put treatment differed from both the 1 mM and 2 mM NH_4_^+^ controls, so the positive effect of Put was not due to an increase in N fertilization (Fig. 11; Supplementary Fig. S4).

## Discussion

### The accumulation of urea cycle intermediates and Put in *M. truncatula* plants under NH_4_^+^ nutrition relates to cation homeostasis and C/N regulation

The accumulation of NH_4_^+^ is considered to be the main biomarker of NH_4_^+^ toxicity (González-Moro *et al.*, 2021), observed in pea (Ariz *et al.*, 2011) lettuce, spinach, lupine (Cruz *et al.*, 2006), tomato (Vega-Mas *et al.*, 2017), wheat (Vega-Mas *et al.*, 2019), *A. thaliana* (Sarasketa *et al.*, 2016), and also for *M. truncatula* plants subjected to high NH_4_^+^ conditions compared with NO_3_^–^-fed plants (Fig. 1). Even though NH_4_^+^-fed *M. truncatula* plants showed the ionic imbalance previously recognized as the main cause of NH_4_^+^ toxicity (Britto and Kronzucker, 2002; Esteban *et al.*, 2016a), they were able to counterbalance K^+^ homeostasis under NH_4_^+^ stress conditions in both shoots and roots. Hence, the maintenance of K^+^ homeostasis may be linked to a greater tolerance towards NH_4_^+^ stress (Zhang *et al.*, 2021), as previously observed in pea plants, which reverse the NH_4_^+^ toxicity when transferred to high-irradiance conditions (Ariz *et al.*, 2011). Pea plants grown under high NH_4_^+^ and high irradiance received a provision of extra C, triggering an ameliorated response to NH_4_^+^ stress by decreasing the Arg pool and increasing Put content, compared with low-irradiance-treated plants (Ariz *et al.*, 2013). Thus, the accumulation of Arg in high-NH_4_^+^-fed *M. truncatula* plants (Fig. 2) may be a response to limited C availability, as observed in plants grown under low irradiance (Ariz *et al.*, 2013). However, *M. truncatula* plants showed the increase in Put content (Fig. 3) that was previously associated with the tolerance response to NH_4_^+^ in high-irradiance-treated plants (Ariz *et al.*, 2013).

The channeling of surplus N into amino acids (Supplementary Fig. S1), particularly the urea-cycle metabolites with low C/N ratio such as Gln, Arg, Orn, and Put, in shoots and roots of *M. truncatula* seedlings grown in high NH_4_^+^ (Fig. 2; Supplementary Fig. S2) reveals a mechanism of N redistribution to cope with high-NH_4_^+^ conditions. The sequestration of NH_4_^+^ excess into low C/N molecules such as Arg, which is the amino acid with the lowest C/N ratio and a major Put precursor, helps plant cells to maintain the endogenous NH_4_^+^ content below toxic levels (Ueda *et al.*, 2008). In addition, the availability of C provided by several anaplerotic reactions is also considered critical in legumes (Ariz *et al.*, 2013) and cereal plants (Vega-Mas *et al.*, 2019). Therefore, there is evidence that the urea cycle is a likely C/N regulatory control point under NH_4_^+^ stress as it represents an NH_4_^+^ sink, as well as a connection between C and N metabolism (Esteban *et al.*, 2016a).

Enhanced tolerance to abiotic stress is usually accompanied by increased contents of PAs in conditions such as salinity, drought, and low/high temperature (Alcázar *et al.*, 2010; Wang *et al.*, 2019), and also under NH_4_^+^ stress (Belastegui-Macadam *et al.*, 2007). Unlike other stress conditions, where Spd and/or Spm have been reported to primarily accumulate ­(Alcázar *et al.*, 2010; Wang *et al.*, 2019), our data indicate that high NH_4_^+^ content induced an increase in Put and, to a lesser extent, Spd, but it did not affect the Spm content in *M. truncatula* roots (Fig. 3). This observation is consistent with the recently proposed adaptive role that Put may play under K^+^ deficiency conditions (Cui *et al.*, 2020), by controlling ion channels and H^+^-ATPases (Pottosin *et al.*, 2021), and it may well explain previous results of studies of plants subjected to NH_4_^+^ stress. Thus, it was described that high irradiance induced the synthesis of Put in high-NH_4_^+^-fed pea plants (Ariz *et al.*, 2013), which maintained or even improved the internal K^+^ content in both shoots and roots (Ariz *et al.*, 2011).

### The role of the urea cycle in connection to PA metabolism

Put has been revealed as an essential metabolite in plant response to stress conditions (Cui *et al.*, 2020). Indeed, the activities of enzymes involved in either Put biosynthesis (Fig. 4) or Put catabolism (Figs 5, 6) were affected by NH_4_^+^. In other types of abiotic stress, Put accumulation resulted from enhanced *ADC* expression (Alcázar *et al.*, 2010). Regarding the synthesis of Put in NH_4_^+^-fed plants, our data showed a switch in the functioning of the urea cycle in roots of NH_4_^+^-fed from the ADC to the ODC pathway (Fig. 4). It seems that this switch to ODC activity is a characteristic of NH_4_^+^ stress in legumes, as the increase in Put in soybean seedlings under NH_4_^+^ has been also reported using radiolabeled Orn (Le Rudulier and Goas, 1977). Indeed, González-Hernández *et al.* (2022) recently suggested a role for the ODC pathway in the NH_4_^+^ tolerance response of tomato plants, since plants overexpressing *ODC* showed improved growth parameters. Interestingly, the NH_4_^+^-sensitive *A. thaliana* does not possess its own *ODC* gene (Hanfrey *et al.*, 2001), which suggests that the ODC enzyme may provide greater plasticity to plants to cope with NH_4_^+^ stress.

Regarding the gene expression levels, except for *MtCPSII1* in roots, all the urea-cycle genes were induced during high-NH_4_^+^ conditions in both shoots and roots (Fig. 9). The remarkable increase in transcript levels of *MtADC1*, *MtADC2*, and *MtODC* in shoots, and *MtADC2* and *MtODC* in roots, highlighted the relevance of the interconnection between the urea cycle and PA metabolism. The strong increase in *ADC* transcript levels in *M. truncatula* plants grown under NH_4_^+^ nutrition contrasted with the decrease in ADC activity. This suggests the existence of post-transcriptional regulatory mechanisms as the described regulation of the ADC enzymes by upstream open reading frames (Jiménez-Bremont *et al.*, 2022). However, we cannot exclude that the reaction catalyzed by ADC was affected by the following AIH and NCPAH activities, as both release NH_4_^+^. In any case, the importance of the pathway has also been reflected in *ODC*-silencing tomato plants under NH_4_^+^ nutrition, which required compensation by *ADC* induction. Furthermore, both *ADC*-silencing and *ODC*-overexpressing mutants showed amelioration of NH_4_^+^ toxicity syndrome (González-Hernández *et al.*, 2022).

Regarding PA catabolism, the lower total activities of the CuAOs confirmed the deceleration of the Put catabolic reactions at a high NH_4_^+^ dose (Fig. 5), and evidenced the existence of a feedback inhibitory mechanism of CuAO by NH_4_^+^ (Fig. 6), as proposed in Esteban *et al.* (2016a). Accordingly, exogenous GABA has been shown to alleviate hypoxia (Wang *et al.*, 2014) and drought damage (Yong *et al.*, 2017) by inducing PA accumulation as well as preventing PA degradation, which supports a feedback regulation of CuAO activity. Therefore, the increase of Put in high-NH_4_^+^-fed plants may be attributed to an increase in ODC activity and a reduction in CuAO activity as the cellular NH_4_^+^ content increases (Fig. 8).

In contrast to the urea-cycle genes, the transcript levels of the PA-catabolism genes showed a general decrease, except for *MtCuAO1* and *MtPAO3* in shoots, and *MtCuAO3* and *MtPAO4* in roots (Fig. 9). In accordance with the reduced CuAO activity observed in 25 mM NH_4_^+^-fed plants, the transcriptional analysis showed that high NH_4_^+^ conditions decreased the predicted apoplastic *MtCuAO2* and *MtCuAO7* in shoots, while the predicted apoplastic *MtCuAO3* was up-regulated in roots of NH_4_^+^-fed plants in comparison to plants grown under NO_3_^–^ nutrition. It is possible that *MtCuAO3* and *MtPAO4* are involved in processes unrelated to the urea cycle–PA metabolism connection (Fig. 10). Indeed, in *A. thaliana*, knockout of the *MtCuAO3* orthologous gene *AtCuAOγ1* contributes to both PA-induced H_2_O_2_ production and nitric oxide biosynthesis involved in the abscisic acid signal transduction pathway (Wimalasekera *et al.*, 2011). Furthermore, the *MtPAO4* orthologous gene in *A. thaliana AtPAO5* participates in plant development and xylem differentiation, interfering with the auxin/cytokinin interplay (Alabdallah *et al.*, 2017).

The activities of AOs could contribute to the reported increase in reactive oxygen species in plants grown under high NH_4_^+^ (Yang *et al.*, 2020) through the production of H_2_O_2_ (Gupta *et al.*, 2016). However, *M. truncatula* plants treated with higher doses of N showed increases in H_2_O_2_ content (Fig. 7), which correlated inversely with the activities of CuAO in both shoots and roots, and with PAO in shoots (Supplementary Fig. S3). Thus, there must exist mechanisms of H_2_O_2_ production other than AOs under NH_4_^+^ toxicity conditions.

### The alleviating effect of Put on *M. truncatula* biomass under NH_4_^+^ nutrition

The internal content of Put has been correlated with the reduction in growth of wheat and pepper plants grown under NH_4_^+^ nutrition (Houdusse *et al.*, 2005), which we also observed in *M. truncatula* plants grown in axenic conditions (Fig. 11; Supplementary Fig. S4). Conversely, in this work we show a remarkable alleviation of NH_4_^+^ toxicity symptoms in *M. truncatula* seedlings when we applied Put early. This effect was similar to the addition of NO_3_^–^ in terms of dry biomass (Houdusse *et al.*, 2005), which was considered a signaling effect (Hachiya *et al.*, 2012). This observation is in agreement with the results of studies of other stresses, where exogenous Put mitigated cadmium (Zhu *et al.*, 2018) and aluminum (Zhu *et al.*, 2019) toxicity in rice, iron deficiency in *A. thaliana* (Zhu *et al.*, 2016), and salinity stress in cucumber (Shu *et al.*, 2012; Yuan *et al.*, 2016, 2019), and also improved drought tolerance in wheat (Doneva *et al.*, 2021). The alleviation by Put of the growth reduction was especially evident during high-stress conditions at 25 mM NH_4_^+^. These results open up the question of how Put mitigates NH_4_^+^ stress, but they also reveal that the common role of Put in the alleviation of stresses works also for NH_4_^+^ toxicity. Furthermore, this function involves a metabolic connection to the urea cycle, highlighting the importance of this route in *M. truncatula*.

### Conclusions

Few studies have dealt with the urea cycle as it has been considered to be incomplete in plants. Our results evidenced that high-NH_4_^+^ conditions increased the transcript levels of genes involved in the urea cycle and the content of intermediates of this cycle. The low C/N ratio intermediates Gln, Arg, Orn, and Put accumulated, evidencing a C limitation under high-NH_4_^+^ conditions. Furthermore, high NH_4_^+^ content altered PA metabolism, leading to a high accumulation of Put, which helps the plant to tolerate the stress. The increase of Put content may be attributed to an increase in ODC activity and a reduction in CuAO activity, probably through an inhibitory effect of NH_4_^+^. Finally, this study shows that exogenously supplied Put leads to alleviation of the growth reduction provoked by NH_4_^+^. The remarkable changes in both the urea cycle and the PA pathway during NH_4_^+^ toxicity provide a clue to its importance as a protection mechanism and as a regulator of C/N metabolism.

## Supplementary data

The following supplementary data are available at *JXB* online.

Table S1. Primers designed for amplification of urea cycle and PA metabolism genes of *M. truncatula* by RT–qPCR.

Table S2. Plant CuAOs and PAOs used for the phylogenetic analysis.

Fig. S1. Effect of different N nutrition on the total amino acid and soluble protein contents.

Fig. S2. Effect of different N nutrition on the amino acid content in shoots and roots of *M. truncatula* seedlings.

Fig. S3. Pearson correlation of the analyzed urea cycle and PA metabolism components.

Fig. S4. Representative image of plants subjected to different N nutrition and supplemented with 0.5 mM Put.

erac235_suppl_Supplementary_Table_S1-S2_Figure_S1-S4

## Data Availability

The data supporting the findings of this study are available from the corresponding author, JFM, upon reasonable request.
