# Supplementary material for: The importance of the urea cycle and its relationships to polyamine metabolism during ammonium stress in Medicago truncatula
Source: J Exp Bot. 2022 May 24;73(16):5581–95. doi: 10.1093/jxb/erac235 (PMC9467648; doi:10.1093/jxb/erac235)
Supplement: erac235_suppl_Supplementary_Table_S1-S2_Figure_S1-S4 [file erac235_suppl_supplementary_table_s1-s2_figure_s1-s4.pdf]

Table S1. Designed primers (5' → 3') for amplifying urea cycle and polyamine metabolism genes and reference genes of *M. truncatula* by RT-qPCR.

	Uniprot ID	Phytozome ID	Forward primer (5' → 3')	Reverse primer (5' → 3')
**Carbamoyl phosphate synthase**				
MtCPSII1	G7JMK3	Medtr4g103830	CAGGCTTTAGGAGGCACGAC	GGCAGTGTAGCAGGGTCAAC
MtCPSII2	G7IU57	Medtr2g093280	CCTTTTCTCTCCAGGCAGCA	TCTGTGGATACAGTCTCGGG
**Ornithine carbamoyltransferase**				
MtOTC1	A0A072VEY9	Medtr1g022420	CTTGGACAGGGCCATTGAGG	ACACAGCATGTCCACCCAAC
MtOTC2	A0A072V483	Medtr3g112050	CCGAATCTCCTCTCACCGAAAA	GTCGAAATGTCCTGTCCCCTG
**Argininosuccinate synthase**				
MtAS1	G7J524	Medtr3g088970	TGATCTAGGCCAAGGGGCAA	CAGGGCGAGCTATTGCAGTT
MtAS2	G7JWM6	Medtr5g042880	TCTGCTCCTTCCCTACGTTCA	CACTGGTTCTTGGCCTCAACT
**Argininosuccinate lyase**				
MtAL	G7J530	Medtr3g100220	ATCACCCCAAGCGACTCTGT	ACACTGGTTCTTTGTCTTCCTGC
**Arginase**				
MtARG	G7JFU5	Medtr4g024960	CGTTCATTGGGAGGGGTTGC	GTCCCGCAAATTCTTGCCCT
**Urease**				
MtURE	G7JC19	Medtr3g085640	GCCATTCACCTGATGGCTCC	AGCAATAACTTCAGTATTAGCCCCA
**Ornithine decarboxylase**				
MtODC	G7J5X8	Medtr3g114870	CCAATTTTGACTGCGCCAGC	ATCTTGTCGGGTGAAACGCC
**Arginine decarboxylase**				
MtADC1	G7J584	Medtr3g113910	TGCCTCAAAGACACTGCAAAC	GGTGAATTGCTCCCAAAGGGT
MtADC2	G7JJX0	Medtr4g072020	TGTAGCGAGAGTGGTAGGGC	CATGAAGCGTAGCAGCCGAT
**Agmatine iminohydrolase**				
MtAIH	G7JT50	Medtr4g112810	GATTGGTTGGCCCGAACGTG	CGCCACCCTGGTAAACACAA
**N-carbamoylputrescine amidohydrolase**				
MtNCPAH	G7ITU5	Medtr2g086600	GGCGCTTCAAGGTGCTGAAA	GAAGCCACGAGAGGTACCAGA
**Copper amine oxidase**				
MtCuAO1	G7ID65	Medtr1g104590	GGTAGCATCAAACTTGGGGTTG	TCAGCTAGCAATGTGCCATGT
MtCuAO2	A0A072V290	Medtr4g117610	TGGTTCCATCAAACCCTCGATTG	CTCCCTTAATCTCATTCGTGTGC
MtCuAO3	G7J7B0	Medtr3g080500	CAAGTGTGGTCTGACAGGGAT	AGGGTAGTCCTCTTGACATGGT
MtCuAO4	G7J4S8	Medtr3g077080	TGGTGAAGCTTTGAATCAGGTTG	TGAGCAAAGTCTCTTCGGCA
MtCuAO5	G7JYY1	Medtr5g033170	GGATTATACGCGCCAGTCCA	ACATCCACCTCAACAACCTGA
MtCuAO6	A0A072TRL3	Medtr8g069505	GGGTTACTCCTTACGCACGC	TGTCACCCCAAATACGTACCAG
MtCuAO7	G7ID64	Medtr1g104550	GTGGCTTGGAGACACATGGAAA	ACACCTGTTAGAGCCACCCCA
**Polyamine oxidase**				
MtPAO1	A0A072UW15	Medtr3g033000	TTTGGACCCAACATCCCCGA	GCTTTGCCTGTGTCAATACCTGC
MtPAO2	G7KD02	Medtr5g090300	CTCGAAGGTCGCCGTCTGTT	TGCAAACTCCATGCAACCATGA
MtPAO3	G7J7X8	Medtr3g064370	TACGTTGCCTGGGGCTTACA	ATCCCTCACTTTCCCTGTCTC
MtPAO4	A2Q567	Medtr2g039160	GGAGCTGGAATGGCAGGCTT	GCTCCCATCTCAATCTTGTCACC
**Reference genes**				
	G7JXI6	Medtr5g022440	TGGCAGGAAAGGGTGTTC	GCCACCTGAATACCAGCAG
	B7FMV8	Medtr3g062450	GATCCAAATTCCCGATGAC	CGGTGGCTTCATACTTGGTC

Table S2. Plant CuAOs and PAOs proteins used for the phylogenetic analysis.

Phylogenetic tree ID	Uniprot ID	Phytozome ID
**Copper amine oxidases**		
AtCuAOa1	P0DO00	At1g31670
AtCuAOa3	F4IAX1	At1g31710
AtCuAOb	O23349	At4g14940
AtCuAOγ1	Q8H1H9	At1g62810
AtCuAOζ	A0A178VYJ2	At2g42490
LcAO	P49252	-
MdAO1	A0A096ZNU5	-
MdAO2	A0A096ZNR5	-
MtCuAO1	G7ID65	Medtr1g104590
MtCuAO2	A0A072V290	Medtr4g117610
MtCuAO3	G7J7B0	Medtr3g080500
MtCuAO4	G7J4S8	Medtr3g077080
MtCuAO5	G7JYY1	Medtr5g033170
MtCuAO6	A0A072TRL3	Medtr8g069505
MtCuAO7	G7ID64	Medtr1g104550
PsAO	Q43077	-
**Polyamine oxidases**		
AtPAO1	Q9FNA2	At5g13700
AtPAO2	Q9SKX5	At2g43020
AtPAO3	Q9LYT1	At3g59050
AtPAO4	Q8H191	At1g65840
AtPAO5	Q9SU79	At4g29720
MtPAO1	A0A072UW15	Medtr3g033000
MtPAO2	G7KD02	Medtr5g090300
MtPAO3	G7J7X8	Medtr3g064370
MtPAO4	A2Q567	Medtr2g039160
OsPAO1	Q5NAI7	Os01g0710200
OsPAO3	Q7X809	Os04g0623300
OsPAO4	Q7XR46	Os04g0671200
OsPAO5	Q0J954	Os04g0671300
OsPAO6	A0A0P0XM10	Os09g0368200
OsPAO7	Q0J290	Os09g0368500

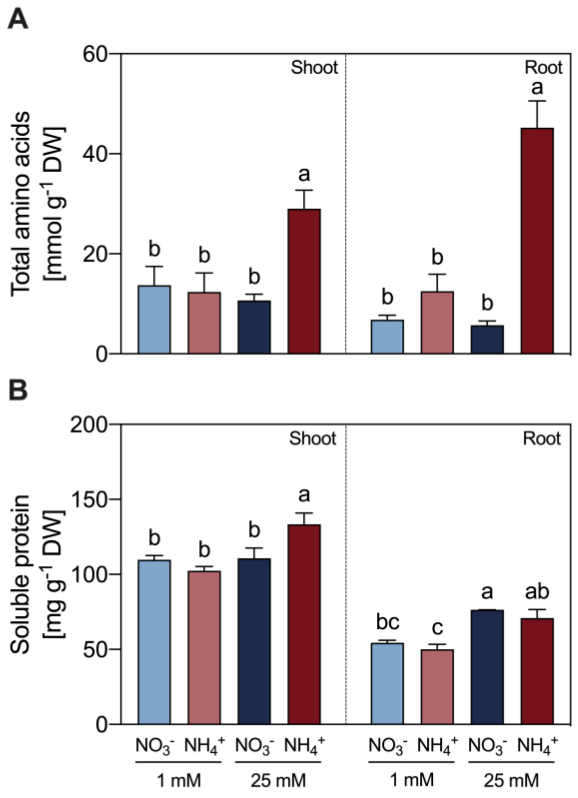

Figure S1. Effect of different N nutrition on the total amino acids (A) and soluble protein (B) contents in both shoots and roots of 14 days-old *M. truncatula* seedlings. Data represent mean values ± S.E (n = 4). Different letters denote statistically significant differences at $\alpha < 0.05$.

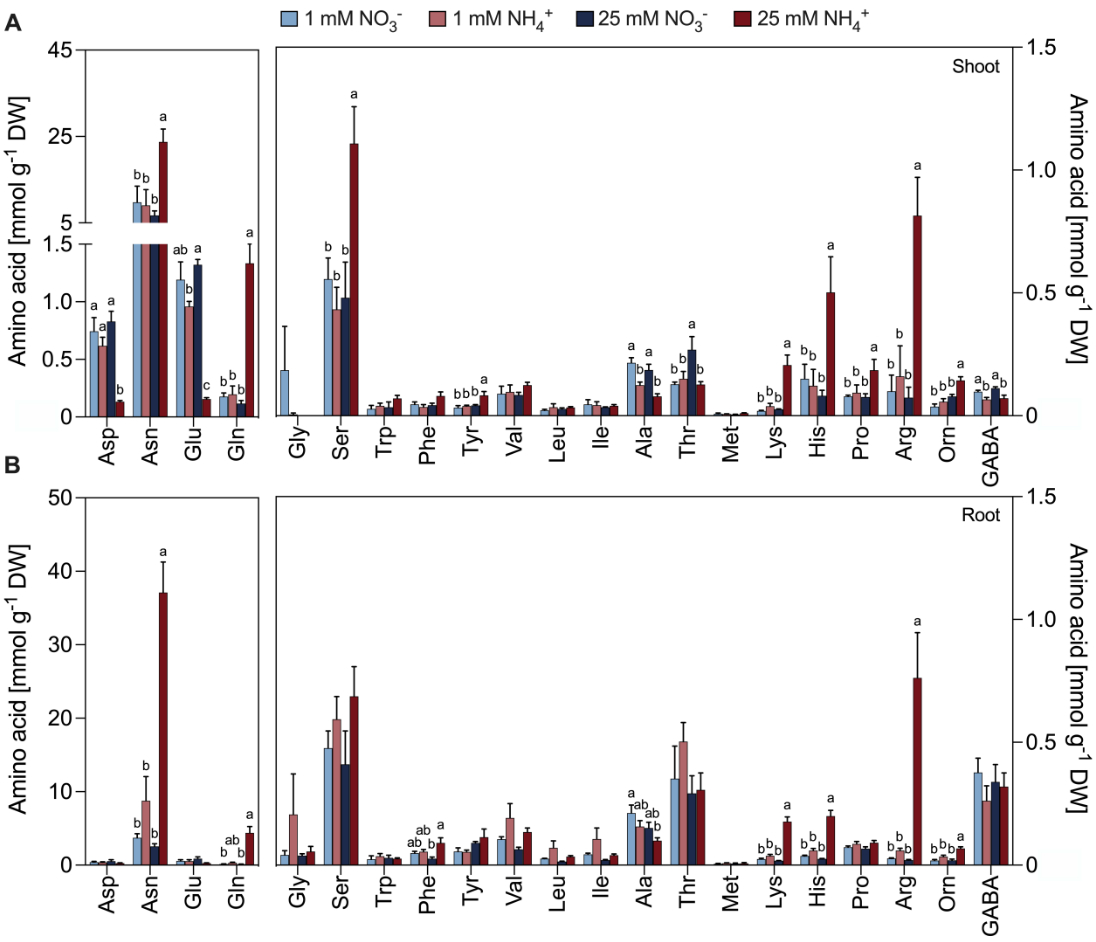

Figure S2. Effect of different N nutrition on the amino acids content in shoots (A) and roots (B) of 14 days-old *M. truncatula* seedlings. Data represent mean values ± S.E (n = 4). Different letters denote statistically significant differences at $\alpha < 0.05$.

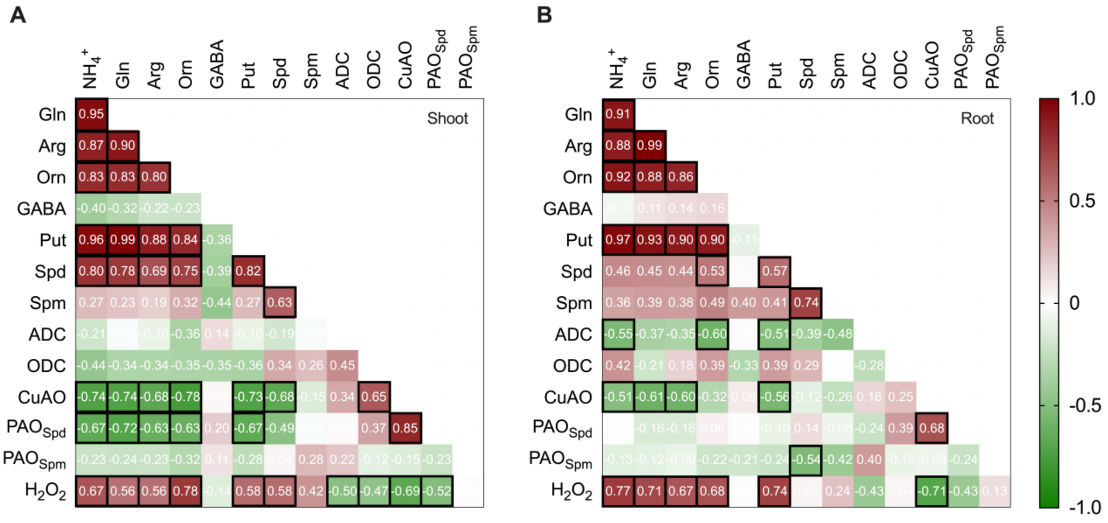

Figure S3. Pearson correlation of the analysed urea cycle and PA metabolism components in shoots (A) and roots (B) of *M. truncatula* seedlings grown under different N nutrition. Black squares denote significant level at α < 0.05.

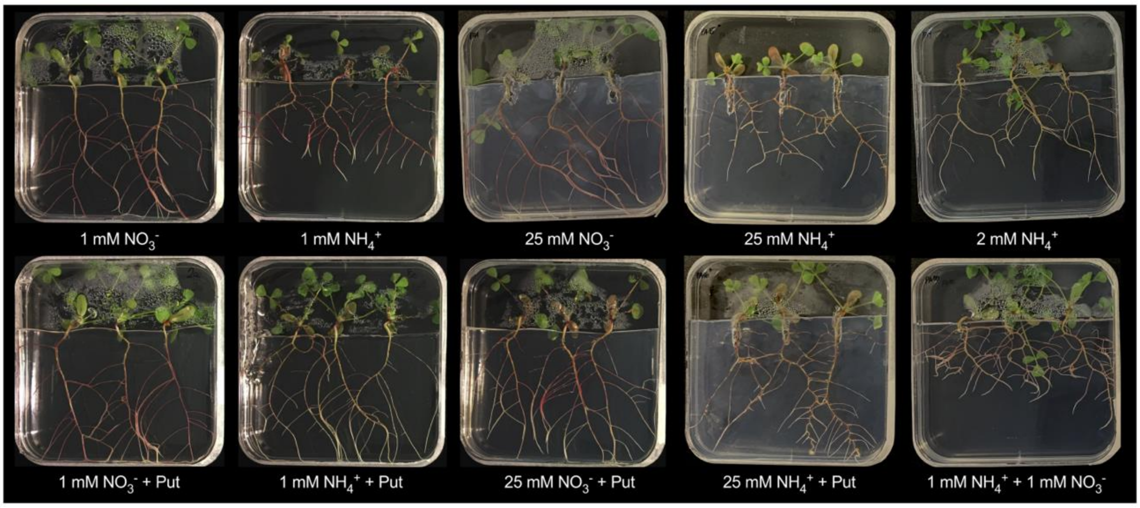

Figure S4. A representative image of plants subjected to different N nutrition and supplemented with 0.5 mM Put.
